# Research on the Performance and Modification Mechanism of Gutta-Percha-Modified Asphalt

**DOI:** 10.3390/polym16131860

**Published:** 2024-06-29

**Authors:** Simeng Yan, Shichao Cui, Naisheng Guo, Zhaoyang Chu, Jun Zhang, Sitong Yan, Xin Jin

**Affiliations:** 1 College of Transportation Engineering, Dalian Maritime University, Dalian 116026, China; 0120180086@dlmu.edu.cn (S.Y.); cuisc@dlmu.edu.cn (S.C.); chzy@dlmu.edu.cn (Z.C.); zhangjun123@dlmu.edu.cn (J.Z.); 2 College of Communication, Tonghua Normal University, Tonghua 134002, China; yansitong@thnu.edu.cn; 3 School of Transportation Engineering, Shenyang Jianzhu University, Shenyang 110168, China

**Keywords:** asphalt, gutta-percha, gutta-percha-modified asphalt, molecular dynamics simulation, preparation process, modification mechanism

## Abstract

Presently, there is a significant focus on the investigation and advancement of polymer-modified asphalt that is both high-performing and environmentally sustainable. This study thoroughly examined the performance and modification mechanism of gutta-percha (GP) as a novel asphalt modifier. The investigation was conducted using a combination of macro- and microscopic testing, as well as molecular dynamics simulations. This work primarily examined the compatibility of GP with asphalt molecular modeling. This paper used molecular dynamics to identify the most suitable mixing temperature. Next, the gray correlation theory was used to discuss the most effective method for preparing gutta-percha-modified asphalt (GPMA). The macro-rheological tests and microscopic performance analysis provided a full understanding of the impact of GP on asphalt properties and the process of alteration. The findings indicate that eucommia ulmoides gum (EUG) exhibits good compatibility with asphalt, while sulfur-vulcanized eucommia ulmoides gum (SEUG) does not demonstrate compatibility with asphalt. Both EUG and SEUG enhance the thermal stability and resistance to deformation of asphalt at high temperatures, with SEUG having a particularly notable effect. However, both additives do not improve the resistance of asphalt to cracking at low temperatures. The manufacturing method for EUG-modified asphalt (EUGMA) involves physical mixing, whereas sulfur-vulcanized eucommia ulmoides gum-modified asphalt (SEUGMA) involves physical mixing together with certain chemical processes. This research establishes a theoretical foundation for the advancement of GP as a novel environmentally friendly and highly effective asphalt modification.

## 1. Introduction

Currently, there is a significant increase in the worldwide demand for asphalt [1]. This has led to ongoing research, development, and design of various types of modified asphalt with enhanced functionality. The selection of the modifier plays a crucial role in determining the performance and application of asphalt. Currently, the majority of polymer modifiers are obtained from the by-products of petroleum cracking [2,3,4], which has environmental pollution hazards and is highly susceptible to price fluctuations influenced by the crude oil market. Hence, the advancement of novel eco-friendly substances as modifiers for asphalt and the advocacy for more sustainable manufacturing processes have emerged as crucial challenges in the realm of worldwide road materials investigation.

Gutta-percha gum, sometimes referred to as eucommia ulmoides gum and barata gum, is a natural polymer substance obtained from the eucommia tree, a tertiary relict species exclusive to China [5,6,7]. The primary chemical component of EUG is trans-1,4-polyisoprene, which is a structural isomer of natural rubber (NR). The molecule’s submethyl groups are evenly distributed on both sides of the C=C bond, resulting in a more symmetrical chemical structure compared to NR. This unusual structure gives rise to a dual rubber–plastic characteristic. In the late 20th century, Yan et al. [8,9,10] introduced the “trans-polyisoprene highly elastic rubber production method”. This method successfully controlled the crystallinity and cross-linking degree of EUG, leading to the development of three distinct forms of polymer plastic materials: a high degree of crystallinity and zero cross-linking degree of the hard plastic materials, a low crystallinity and low cross-linking degree of the thermoplastic materials, and amorphous, rubbery materials at critical cross-linking levels. EUG, with its excellent crystallinity and unique dual rubber–plastic properties, has been developed for a variety of innovative applications, including green tires, vibration- and sound-absorbing materials, shape memory materials, self-healing materials, interfacial compatibilizers, high-performance composite films, elastomers, and biodegradable composites. These technological advances have not only enriched the research field of materials science, but also promoted the application and development of EUG in the fields of textiles, aerospace, biomedicine, transportation, sports, and construction. Especially in the field of pavement materials, many researchers have taken EUG as an asphalt modifier as a cutting-edge topic for in-depth study to provide new solutions for realizing the goal of sustainable development [11,12].

Li et al. [13] were the first to demonstrate that SEUG as an asphalt modifier may greatly enhance the high-temperature performance of asphalt and to some degree increase its low-temperature performance. They confirm the viability of using EUG in asphalt modification. Later, Li et al. [14] discovered that EUG had excellent dispersion in asphalt, and SEUG could significantly enhance the high- and low-temperature characteristics of asphalt. Li et al. [15] employed a solution-grafting technique to attach maleic anhydride onto EUG. The outcomes demonstrated that the grafted EUG facilitated the formation of a connecting channel between SBS (styrene–butadiene–styrene block copolymer) and matrix asphalt (BA), hence enhancing the compatibility at the interfaces. Fang et al. [16] demonstrated that the addition of SEUG may greatly enhance the performance of asphalt at low temperatures. The optimal proportion of SEUG in asphalt is determined to be 5%, while the optimal proportion of sulfur in EUG is also 5% [17]. In a further investigation, Chen et al. [17] determined that the optimal performance of SEUG-modified asphalt (SEUGMA) occurs when the sulfur concentration is within the range between 4wt% and 5wt% of EUG. Li et al. [18] conducted research to intensify the chemical reaction between rubber powder and asphalt. They achieved this by grafting maleic anhydride EUG onto the rubber powder through dry mixing. The results demonstrated that the grafted maleic anhydride EUG created chemical cross-linking between the rubber powder and asphalt, resulting in the formation of a three-dimensional network structure in the rubber asphalt. This improved the performance of the rubber asphalt and achieved the desired effect of modifying it with a vitamin oxide connecting agent (TOR). Deng et al. [19,20] conducted further extensive investigations and confirmed this finding. Li et al. [21] demonstrated that the addition of SEUG enhances the performance of asphalt under both high- and low-temperature conditions. The optimal amount of SEUG is 10wt%. Furthermore, high-temperature and high-speed shearing facilitate the compatibility between SEUG and asphalt. Cui et al. [22] synthesized SEUGMA using the melt-blending technique. The study revealed that the optimal weight percentage of EUG was 3.5wt%, the optimal weight percentage of sulfur was 6wt%, the optimal shear time was 45 min, and the optimal development time was 120 min. Du et al. [23] determined that the minimum polymerization degree of EUG is 35, while the minimum polymerization degree of natural rubber (NR) is 20. They also found that the solubility parameter of EUG is 16.6 (J/cm^3^)^1/2^, and the solubility parameter of NR is 16.2 (J/cm^3^)^1/2^. By integrating MD and experimental results, they concluded that the compatibility between EUG and NR is reasonable. These research findings offer a crucial theoretical foundation for the industrialized manufacturing of GPMA.

While existing studies have yielded a series of fruitful outcomes, the research on the impact of EUG vulcanization and non-vulcanization preparation techniques on the properties of GPMA and its modification mechanism is still limited to a broad perspective. This paper used molecular simulation and experimentation to investigate the optimization of characteristics and interaction mechanisms of GPMA to expand the research scope. This paper employed the molecular dynamics approach to initially create molecular models of BA and GPMA with varying compositions. The compatibility of GP and asphalt molecules at the molecular level was analyzed using molecular dynamics modeling. Key indicators such as the Hansen solubility parameter (*δ*_Hansen_) and interaction energies were estimated for this purpose. Additionally, the appropriate mixing temperature was found. This study investigates the most effective method of preparing GPMA using the gray correlation theory and validates the trustworthiness of the molecular simulation findings. In conclusion, the macro-rheological tests and microscopic performance analysis provide a full understanding of the performances of GPMA and its modification mechanism.

## 2. Molecular Modeling and Verification

### 2.1. Molecular Modeling

#### 2.1.1. BA Molecular Modeling

**Figure 1:**
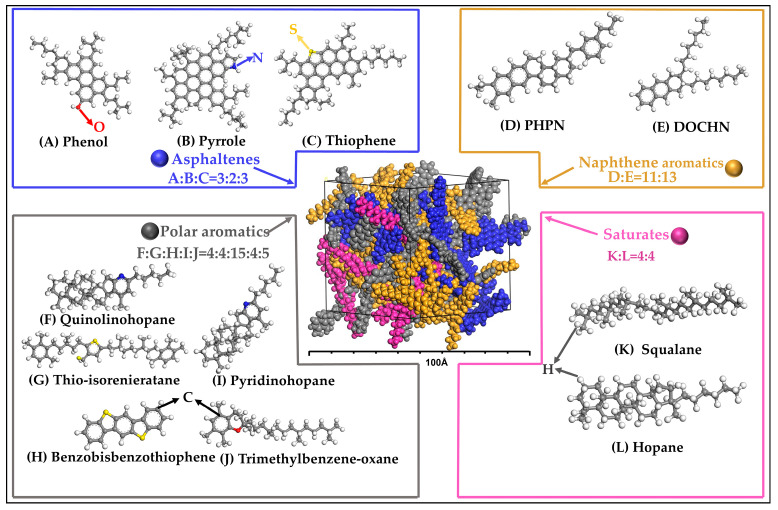
The four-component, twelve-class molecular AAA-1 model of base asphalt.

The development of molecular models of asphalt is key to understanding and predicting asphalt properties. Historically, the development of molecular models of asphalt progressed through a series of stages from simple three-component systems to more complex four-component systems, and ultimately to four-component models containing 12 molecules [24,25]. Studies have confirmed that the four-component 12-class molecular model is more advantageous in simulating the properties of real asphalt materials [26,27]. For this reason, the AAA-1 model (the AAA-1 model is a molecular dynamics model of base asphalt proposed by Li and Greenfield [24,25]) was selected as the BA molecular model in this paper, and the schematic diagrams of asphalt components and structures are shown in Figure 1.

#### 2.1.2. EUG Molecular Modeling

**Figure 2:**
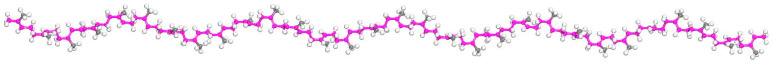
Molecular model of EUG.

EUG is mostly composed of homopolymers that are formed through the polymerization of trans-1,4-polyisoprene monomers. The degree of polymerization (*N*) of a homopolymer is a crucial parameter for identifying the physicochemical properties of polymeric materials [28]. Based on the literature research, it was discovered that the minimal degree of polymerization for EUG is *N* = 30 [29,30]. Thus, in this study, a prototypical molecular model of EUG with a polymerization degree of 30 was created using MS software version 2005 employing the required repeating unit technique (RUM). The molecular structure is visually depicted in Figure 2.

#### 2.1.3. SEUG Molecular Modeling

**Figure 3:**
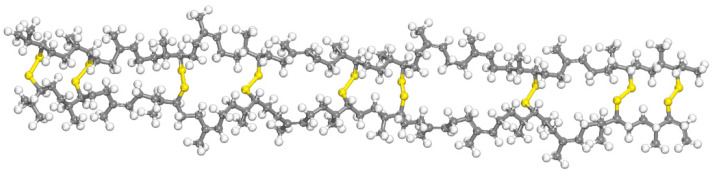
The SEUG molecular model with a cross-linking degree of 60%: Yellow represents the C-S-S-C disulfide bridges.

The SEUG model was developed from the EUG molecular model by using C-S-S-C groups as cross-linking bonds. Our group discovered that the ideal cross-linking degree for SEUG should be maintained 40wt%~80wt% [29]. In this work, the degree of cross-linking (DC) of SEUG was chosen to be 60wt%. A molecular model representing this degree of cross-linking is depicted in Figure 3. Based on the literature research [31,32], the cross-linking degree was calculated as

(1) $$DC=\frac{2N_{CL}}{N_{mono}}\cdot 100\%$$

where *N_CL_* denotes the total number of crosslinked bonds and *N_mono_* denotes the number of monomers.

**Figure 4:**
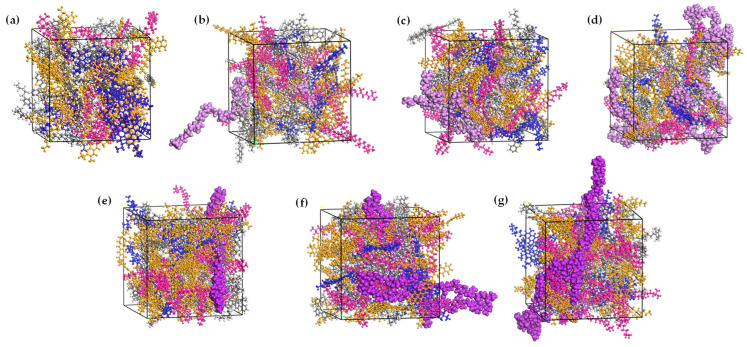
BA and GPMA molecular crystal cell models:(**a**) BA molecular model; (**b**) 5wt% EUGMA molecular model; (**c**) 10wt% EUGMA molecular model; (**d**) 15wt% EUGMA molecular model; (**e**) 5wt% SEUGMA molecular model; (**f**) 10wt% SEUGMA molecular model; (**g**) 15wt% SEUGMA molecular model. In Figure 4, light purple represents the molecular model of EUG, and dark purple represents the molecular model of SEUG.

**Figure 5:**
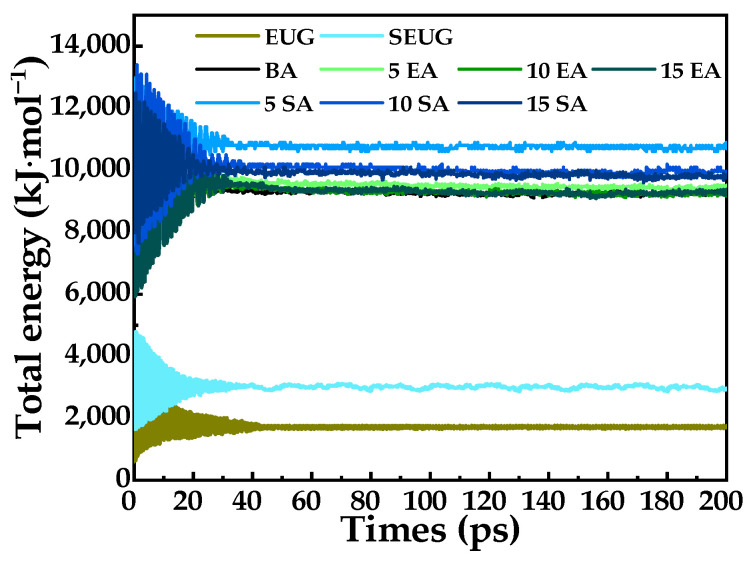
This curve shows how energy changes over time in molecular dynamics simulations run with the NPT ensemble using the GP and asphalt models.

Investigations in the literature have found that the typical concentration range of GPMA is 5% to 15% [21,29]. In this study, three molecular models of EUG and SEUG were incorporated into the BA model, each with a reasonable conformation in the dry state. The GP molecules were added in proportions of 5wt%, 10wt%, and 15wt% of the asphalt mass, denoted as XEA/SA, where X represents the respective contents. The molecular dynamics simulation method proceeded as follows: Initially, molecular models of BA and GP were constructed using MS software, as shown in Figure 4. Afterward, the GP and BA molecules were enclosed within the GPMA cell model using the amorphous cell module. Subsequently, the smart method was employed to optimize the structure, resulting in a molecular model that possesses optimal local energy states [33,34]. Afterward, the model underwent an annealing process where it was heated from 25 °C to 1527 °C and then cooled back to 25 °C in 10 gradual increments. This process was repeated five times in order to remove any structural flaws and areas of high stress [35]. Next, the asphalt model underwent the initial kinetic equilibrium at 200 ps under canonical ensemble (NVT) conditions to simulate the equilibrium process of asphalt in the mixing state [36,37]. Finally, the second kinetic equilibrium, lasting 200 ps, was conducted under constant pressure and temperature (NPT) conditions to ensure the accuracy of the simulation results. During this time, the BA molecular model and the GPMA molecular model with varying contents were used, as depicted in Figure 4. Additionally, the total energy versus time curve can be observed in Figure 5. Figure 5 illustrates that the total energy of the system had a quick adjustment phase within the initial 40 ps, followed by a gradual stabilization. It signifies that all systems have achieved thermodynamic equilibrium after two molecular dynamics simulations, establishing a strong basis for subsequent GMPA performance analysis and material design.

### 2.2. Validation in Molecular Modeling

The density (*ρ*) and Hansen solubility parameter (*δ*_Hansen_) are crucial thermodynamic indicators used to evaluate the precision of molecular models. Table 1 provides comprehensive thermodynamic parameter data for all chemical models following the attainment of thermodynamic equilibrium. *ρ* is determined by averaging the density from the last 50 picoseconds of the equilibrium portion of the 200-picosecond trajectory ran at a temperature of 25 °C. The *ρ* and *δ*_Hansen_ values obtained from the simulation exhibit a deviation of less than 5% from the values reported in the literature or measured. Although there is some deviation, the simulation results still align well with the literature or measured results. This suggests that the current molecular models and force fields adequately describe the asphalt system and can be employed to investigate the properties of asphalt materials at a molecular level.

## 3. Determination of Test Materials, GPMA Preparation Process, and Design of Test Program

### 3.1. Test Materials

#### 3.1.1. Base Asphalt

The investigation selected Liaohe A-90 road petroleum asphalt as the BA, and its technical specifications may be identified in Table 2.

#### 3.1.2. EUG

The investigation applied natural commercial eucommia ulmoides gum (EUG) particles. Previous research has shown that the size of rubber particles has a notable impact on asphalt properties. Specifically, smaller rubber particles have been found to enhance the high-temperature stability, fatigue resistance, and storage stability of asphalt [44,45]. In this work, an ultra-low-temperature pulverizer (model BCF-450) was applied to convert EUG into a 200-mesh powder. The objective was to enhance the interaction between EUG and asphalt, hence enhancing the performance of the asphalt.

#### 3.1.3. Additives

The additives used in this investigation comprised sulfur, zinc oxide, stearic acid, N-cyclohexyl-2-benzothiazolyl subsulfonamide, silica nanoparticles, naphthenic oils, and epoxy resins. It is important to note that all of these additives were analytically pure.

### 3.2. GPMA Preparation

To distinguish between them, this investigation defined nonvulcanized eucommia ulmoides gum-modified asphalt as EUGMA and vulcanized eucommia ulmoides gum-modified asphalt as SEUGMA, and both are collectively known as gutta-percha-modified asphalt (GPMA).

#### 3.2.1. SEUG Preparation Process

**Figure 6:**
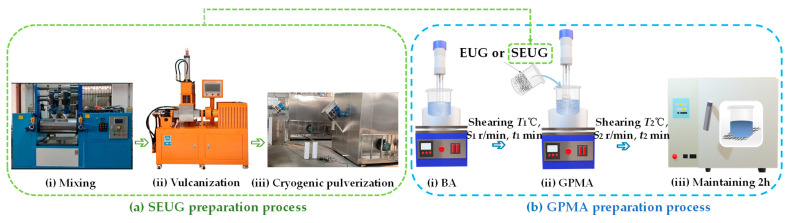
GPMA preparation process flow chart.

The SEUG preparation process is illustrated in Figure 6a according to the research findings of group [46]. Initially, the double-roll opener was heated to a temperature range of 70–80 °C and maintained at that temperature for a duration of 5 min. Afterward, pre-weighed EUG powder was added to the machine in separate batches to ensure even distribution. Once the EUG was heated and softened and mixed thoroughly, the double-roll spacing was modified, and zinc oxide, stearic acid, nano-silicon dioxide, naphthenic oil, epoxy resin, N-cyclohexyl-2-benzothiazolyl subsulfonamide, and sulfur were sequentially incorporated. Subsequently, the mixture was heated to approximately 70 °C and stirred for a duration of 3 to 5 min. Subsequently, the uniformly blended samples were subjected to vulcanization on a plate vulcanizing machine, with a temperature of 150 °C, a pressure of 120 MPa, and a duration of 35 min. Following the vulcanization process, the SEUG modifier samples were transformed into powder form using low-temperature pulverization technology for future applications.

#### 3.2.2. Preparation Process of GPMA

The research involved the preparation of GPMA using the melt-blending method, as illustrated in Figure 6a,b. The precise procedure is as follows: Initially, the BA was heated until it became molten. The initial rotor speed of the shear was set to S1 revolutions per minute; the temperature was set to *T*_1_ °C, and the shear was cut for *t*_1_ min. This was performed to ensure that the asphalt had the appropriate fluidity for the dispersion of the modifier. The GPMA powder, measured by weight, was added to the asphalt in batches. Each batch was added with a 2 min interval. The rotor speed of the shear was then gradually increased to *S*_2_ r/min, and the shear temperature was set to *T*_2_ °C. The shear process continued for *t*_2_ min until the GPMA was evenly distributed in the asphalt. Ultimately, the GPMA, which was thoroughly blended to achieve a uniform composition, was fabricated in an oven set at a temperature of 180 °C for a duration of 2 h in order to remove any trapped air bubbles.

#### 3.2.3. Determination of the Optimum Preparation Process for GPMA

Previous investigations have demonstrated that the shear temperature (*T*), shear rate (*v*), and shear time (*t*) exert substantial influence on the diffusion, compatibility, and physical and chemical characteristics of asphalt and modifier [47,48,49]. In this study, the experimental variables *T*, *v*, and *t* were used, and the L_9_(3^4^) orthogonal table was used to design the experimental program for optimizing the preparation process of GPMA. The specific parameters can be found in Table 3.

**Figure 7:**
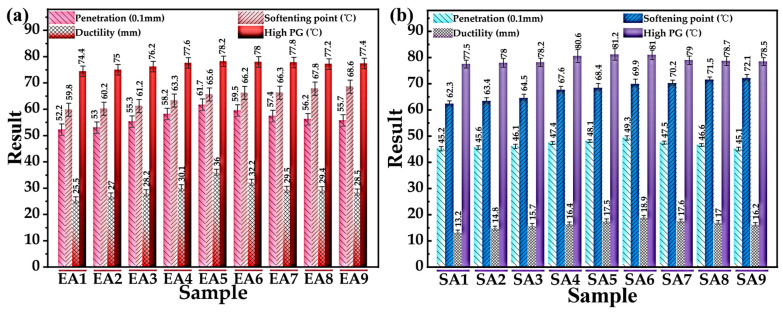
Results of basic physical properties and high-temperature grading temperatures of GPMA: (**a**) EUGMA and (**b**) SEUGMA.

To determine the optimal preparation process of GPMA, the 18 kinds of GPMA samples obtained from Table 3 were subjected to the basic physical properties and PG continuous grading test, with a uniform GP content of 10wt%; the relevant test results are shown in Figure 7. As seen from Figure 7, the basic physical properties of GPMA under different preparation processes show a differentiated pattern, which indicates that the shear temperature, the shear rate, and the shear time had a significant effect on the properties of GPMA, among which the basic physical properties of EA5 and SA5 are better, but the determination of the optimal preparation process parameters of GPMA still needs to be further discussed.

In order to identify the most effective method for preparing GPMA, the 18 samples of GPMA obtained from Table 3 were analyzed for their basic physical properties and PG continuous grading test. The samples have a consistent GP content of 10wt%. The results of these tests are presented in Figure 7. Figure 7 demonstrates that the fundamental physical characteristics of GPMA vary depending on the preparation methods. This suggests that the temperature, rate, and duration of shearing have a notable impact on the properties of GPMA. Specifically, the basic physical properties of EA5 and SA5 are superior. However, further investigation is required to determine the optimal preparation process parameters for GPMA.

The gray correlation theory was applied to assess the level of correlation among various preparation processes. The procedure for calculating was as follows: We chose the highest values of penetration, softening point, 5 °C ductility, and high-temperature continuous grading temperature as the reference sequence. We used the remaining test results as the comparison sequence. The comparative sequence was made dimensionless in order to mitigate the influence of varying magnitudes. The absolute value of the discrepancy between the dimensionless comparison series and the reference sequences was computed to create the difference sequences. The correlation coefficient and the degree of correlation were determined by applying Equations (2) and (3), and the specific results are provided in Table 4.

(2) $$\xi_{i}(k)=\frac{\min_{i}\min_{k}\Delta_{i}(k)+\rho \max_{i}\max_{k}\Delta_{i}(k)}{\Delta_{i}(k)+\rho \max_{i}\max_{k}\Delta_{i}(k)}$$

(3) $$\gamma_{i}(k)=\frac{1}{n}\sum_{k=1}^{n}\xi_{i}(k)$$

where $\xi_{i}(k)$ represents the correlation coefficient; $\min_{i}\min_{k}\Delta_{i}(k)$ represents the minimum difference between the two levels of the difference series; $\max_{i}\max_{k}\Delta_{i}(k)$ represents the largest difference in the two levels of the difference series; ρ=0.5;$\gamma_{i}(k)$ represents the degree of correlation.

As shown in Table 4, among the EUGMA experimental programs, the EA5 program has the largest, indicating that the optimal preparation process parameters of EUGMA are *T*_1_ = 145 °C, *T*_2_ = 165 °C; *S*_1_ = 3000 r/min, *S*_2_ = 5000 r/min; and *t*_1_ = 60 min, *t*_2_ = 60 min. Similarly, the optimal preparation process parameters of SEUGMA are *T*_1_ = 155 °C, *T*_2_ = 180 °C; *S*_1_ = 4000 r/min *S*_2_ = 6000 r/min; and *t*_1_ = 90 min, *t*_2_ = 90 min. Among them, the secondary shear temperature *T*_2_ for EUGMA and SEUGMA were consistent with the MS simulation results, which proved that the simulation results of MS were true and reliable. Therefore, all subsequent GPMA were prepared by the above optimal preparation process to exclude the potential interference of the preparation process on the test results.

### 3.3. Design of Experiments

#### 3.3.1. Dynamic Shear Rheology (DSR) Test

In this research, DSR (Kinexus Prime, NETZSCH Co., Selb, Bayern, Germany) was utilized for the testing of high-temperature rutting resistance of asphalt in the mode of temperature scanning, with a test temperature of 46~82 °C, a temperature gradient of 6 °C, a gap plate with a diameter of 25 mm and a gap of 1 mm, a control strain of 12%, and a loading frequency of 10 rad/s.

#### 3.3.2. Multiple Stress Creep Recovery (MSCR) Test

The MSCR test is a test of asphalt high-temperature creep recovery performance by DSR. The stress recovery mode was adopted; the test temperature was 64 °C, and the stresses of 0.1 kPa and 3.2 kPa were applied with loading of 1 s and unloading of 9 s as a cycle, respectively, and each level of stress was loaded for 10 times in a cycle, and the irrecoverable creep softness (*J*_nr_) was measured along with the creep recovery rate (*R*).

#### 3.3.3. Low-Temperature Bending Beam Rheology (BBR) Test

The low-temperature bending rheology test of asphalt was performed using BBR (CANNON Instrument Co., Bakersfield, CA, USA) to measure the low-temperature creep properties of asphalt at −12~−24 °C with a temperature gradient of 6 °C. Creep stiffness and creep rate of asphalt specimens were obtained when the loading time was 60 s.

#### 3.3.4. Fourier Transform Infrared Spectroscopy (FTIR) Test

In this research, FTIR (Nicolet iS20, Thermo Fisher Co., Waltham, MA, USA) was employed to measure and analyze the characteristic peaks of the specimens. The test mode was ATR mode with a wave number range of 400 cm^−1^~4000 cm^−1^, and the number of scans was 32 times with a resolution of 4 cm^−1^.

#### 3.3.5. Fluorescence Microscopy (FM) Test

In this research, we observed the compatibility between asphalt and GP by FM (Ckx53, OLYMPUS Co., Tokyo, Japan). Samples were prepared by the “glass slide method”. Under short-wavelength light irradiation, GP emits fluorescence, while the asphalt phase does not. Therefore, the asphalt phase and the GP phase can be recognized by FM images, allowing for non-destructive detection of the polymer phase distribution within the asphalt.

#### 3.3.6. Scanning Electron Microscope (SEM) Test

In the present research, SEM (S-3000N, Hitachi Co., Tokyo, Japan) was utilized to observe the microscopic morphology of asphalt. Since both GP and asphalt are non-conductive materials, the asphalt samples were firstly dried in a carbon dioxide critical point dryer, then sputter-coated with gold using a surface treatment machine, and finally observed and analyzed by SEM with a magnification of 500 times.

#### 3.3.7. Atomic Force Microscopy (AFM) Tests

In this research, an AFM (MultiMode 8, BRUKER Co., Billerica, MA, USA) was utilized for the observation of asphalt microstructure. The test temperature was 25 °C; the scanning mode was peak force tapping mode; the vibration frequency was 1 kHz; the scanning area was 40 μm × 40 μm; and the resolution was 512 × 512.

## 4. Results and Discussion

### 4.1. Compatibility of GPMA with Asphalt

To investigate the compatibility between asphalt and GP, the Hansen solubility parameter (*δ*_Hansen_) and the interaction energy were used as indicators for qualitative and quantitative evaluation in this work, respectively.

#### 4.1.1. Hansen Solubility Parameter

*δ*_Hansen_ was calculated by analyzing the *CED* and solubility parameters of the trajectory file according to Equations (4) and (5) [50]. *δ*_Hansen_ quantifies the compatibility between asphalt and GP from the three dimensions of van der Waals forces, electrostatic interactions, and hydrogen bonding, which is more reflective of the true properties of the material than the single use of *δ*_total_ or *δ*_V_ as the evaluation index.

(4) $$CED=\frac{E_{coh}}{V}$$

(5) $$\delta_{\mathrm{Hansen}}=\sqrt{CED}=\sqrt{\delta_{V}^{2}+\delta_{\varepsilon }^{2}+\delta_{o}^{2}}$$

where *CED* is the cohesive energy density; *E_coh_* is the cohesive energy; *V* is the molar volume; $\delta_{V}$, $\delta_{\varepsilon }$, and $\delta_{o}$ are van der Waals, electrostatic and other solubility parameters, respectively.

**Figure 8:**
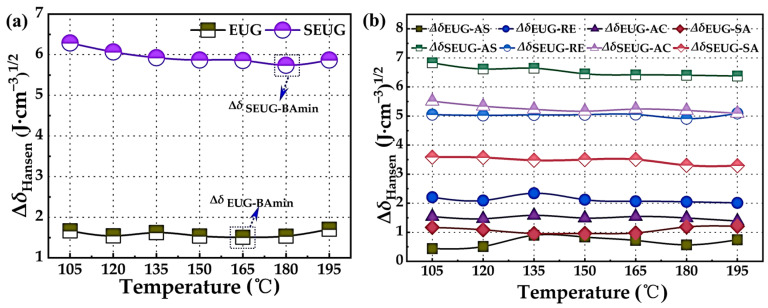
The solubility parameters and differences in solubility parameters of the BA and GP molecular models: (**a**) the overall solubility parameter difference between the BA and GP; (**b**) the solubility parameter difference between the four components of the BA and GP. In Figure 8, AS denotes asphaltenes; RE denotes polar aromatics; AC denotes naphthene aromatics; SA denotes saturates.

Figure 8 shows the variation in Δ*δ*_Hansen_ with temperature for base asphalt as Figure 8a and its four components as Figure 8b with GP. From Figure 8a, it can be seen that the Δ*δ*_Hansen_ between EUG and asphalt is less than 4.10 (J·cm^−3^)^1/2^ at different temperatures, meeting the specification requirements. According to the principle of similar solubility parameters, it indicates that the EUG and asphalt are compatible with each other. From Figure 8b, it can be seen that the asphaltene and saturate in the four components of EUG and asphaltene are smaller, indicating that EUG and asphaltene and saturate of the compatibility is better. This is due to the fact that asphaltene has strong polarity and EUG also has a certain polarity. According to the principle of similar compatibility, EUG is able to form a stable micellar system with asphaltenes and saturates through the polar interaction. Especially at 165 °C, the values between EUG and asphaltenes and saturates reached a very small value, which indicated that the homogeneity of EUGMA system reached the best, and it can be considered that the preparation of EUGMA can be carried out at this temperature.

In contrast, the Δ*δ*_Hansen_ between SEUG and asphalt are generally greater than 4.10 (J·cm^−3^)^1/2^, indicating that the compatibility between SEUG and asphalt is poor. However, the overall and four-component compatibility between SEUG and asphalt reached a minimum at 180 °C, indicating that the compatibility between SEUG and asphalt reached the optimal at this time, which is due to the fact that under high-temperature conditions, the crosslinked structure of SEUG forms intermolecular interactions with naphthene aromatics and polar aromatics in the asphalt through the π-π conjugation, but such interactions are facilitated by the temperature stresses, which are weak and unstable, and in order to promote the miscibility of SEUG and asphalt, it can be considered that at this temperature, it is possible to use the asphalt in a more efficient way, so that the compatibility of SEUG and asphalt can be improved. In order to promote the miscibility between SEUG and asphalt, the preparation of SEUGMA can be considered at this temperature.

#### 4.1.2. Interaction Energies

The essence of compatibility lies in the interaction between different molecules in a co-mingled system or between a molecule and an external field. The strength of the interaction is often quantified using the metric of interaction energy. The lower the interaction energy, the better the intermolecular compatibility and the more stable the structure of the blend system. In the case of systems j and k, for example, the interaction energy can be calculated by the formula

(6) $$E_{p}=E_{jkp}-E_{jp}-E_{kp}$$

(7) $$E_{V}=E_{jkV}-E_{jV}-E_{kV}$$

(8) $$E_{\varepsilon }=E_{jk\varepsilon }-E_{j\varepsilon }-E_{k\varepsilon }$$

where $E_{p}$, $E_{V}$, and $E_{\varepsilon }$ denote the non-bonding potential energy, van der Waals potential energy, and electrostatic potential energy of the j and k systems, respectively; $E_{jkp}$, $E_{jp}$, and $E_{kp}$; $E_{jkV}$, $E_{jV}$, and $E_{kV}$; $E_{jk\varepsilon }$, $E_{j\varepsilon }$, and $E_{k\varepsilon }$ denote the non-bonding potential energy, van der Waals potential energy, and electrostatic potential energy of the jk co-mingled system, the j system, and the k system, respectively.

**Figure 9:**
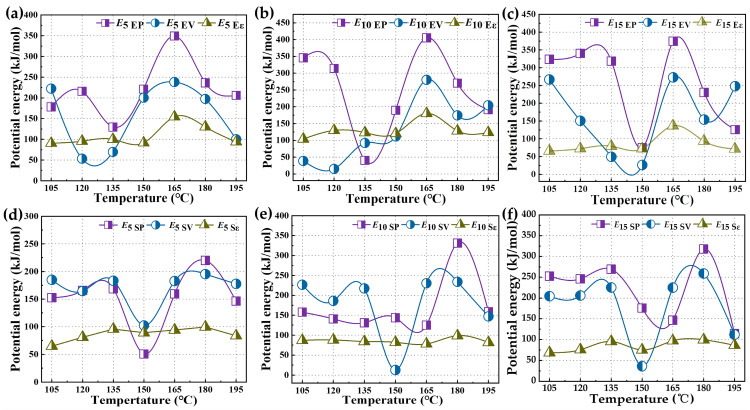
The molecular potential energy of GPMA: (**a**) potential energy of 5wt% EUGMA, (**b**) potential energy of 10wt% EUGMA, (**c**) potential energy of 15wt% EUGMA, (**d**) potential energy of 5wt% SEUGMA, (**e**) potential energy of 10wt% SEUGMA, and (**f**) potential energy of 15wt% SEUGMA.

Figure 9 shows the interaction energies between asphalt and EUG and SEUG molecules with different proportions. From Figure 9, it can be found that the interaction energies between EUG and SEUG and asphalt molecules are mainly attributed to the contribution of van der Waals energy. Specifically, the interaction energy between EUG and asphalt molecules with different proportions peaks at 165 °C, while the interaction energy between SEUG and asphalt molecules peaks at 180 °C. This phenomenon may be due to the volume expansion of the blends due to the increase in temperature, which leads to an increase in the distance between the modifier molecules and asphalt molecules, thus causing the original repulsive force to be transformed into a gravitational force. In addition, the interaction energy between EUG and asphalt molecules was larger compared to that between SEUG and asphalt molecules, indicating a stronger interaction between EUG and asphalt molecules. This finding is consistent with previous conclusions based on solubility a parameter analysis.

### 4.2. Rheological Properties of GPMA

#### 4.2.1. High-Temperature Rutting Resistance

**Figure 10:**
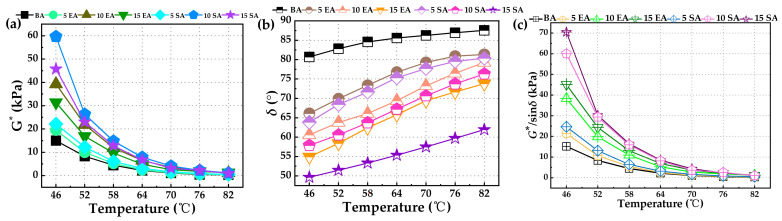
The rutting resistance of BA, EUGMA, and SEUGMA at high temperature: (**a**) G* of BA and GPMA; (**b**) *δ* of BA and GPMA; (**c**) G*/sin*δ* of BA and GPMA.

The complex shear modulus (G*) quantifies the ability of asphalt to resist shear deformation. The phase angle (*δ*) indicates the proportion of the viscoelastic component of the asphalt. G*/sin*δ* represents the asphalt’s resistance to rutting at high temperatures. A higher value of G* indicates a smaller value of *δ*, which in turn indicates stronger resistance to shear deformation. Therefore, a larger value of G*/sin*δ* indicates better resistance to rutting [51]. Figure 10 displays the rules of variation for G*, *δ*, and G*/sin*δ* across various types of asphalt.

Figure 10 illustrates that as the temperature rises, every type of asphalt exhibits a gradual decrease in G*, a gradual increase in *δ*, and a gradual decrease in G*/sin*δ*. This phenomenon can be attributed to the progressive softening of asphalt as the temperature rises, causing the elastic properties to transition into the viscous properties. This transition weakens the asphalt’s ability to recover from deformation and consequently reduces its resistance to high-temperature rutting [52,53]. At the identical temperature, both EUGMA and SEUGMA exhibited larger G*, smaller *δ*, and larger G*/sin*δ* in comparison to BA. These findings demonstrate that GP greatly improves the ability of asphalt to resist rutting at high temperatures. Furthermore, the degree of improvement becomes increasingly significant as the amount of modifier used increases. The observed phenomenon can be explained by the absorption of light components in asphalt by GP, which leads to a swelling reaction. As a result, the elastic properties of asphalt are increased while the viscous properties are decreased, thereby improving the ability to resist deformation of asphalt. The G* of SEUGMA was higher than that of EUGMA under identical temperature and content conditions, particularly when the content reached 15wt%. At this point, both the G* and the G*/sin*δ* of SEUGMA reached their maximum values, while the *δ* decreased to its minimum. This indicates that the presence of a more abundant crosslinked network structure in SEUG, as compared to EUG, can significantly improve the high-temperature performance and elastic recovery ability of BA. Thus, the 15wt% SEUGMA sample exhibited superior high-temperature performance.

#### 4.2.2. High-Temperature Creep Recovery Performance

MSCR has the ability to accurately represent the deformation response and delayed elastic properties of asphalt. This paper examined two stress levels, specifically 0.1 kPa and 3.2 kPa. The purpose of the 0.1 kPa stress level is to replicate the creep behavior of the pavement under lightly loaded traffic conditions. Conversely, the 3.2 kPa stress level is intended to simulate the creep characteristics of the pavement in a heavily loaded traffic environment. For a given loading time, as the cumulative strain value of asphalt decreases, the unrecoverable creep flexibility (*J_nr_*) also decreases, and the elastic recovery rate (*R*) increases. This suggests that the asphalt is more resistant to permanent deformation.

**Figure 11:**
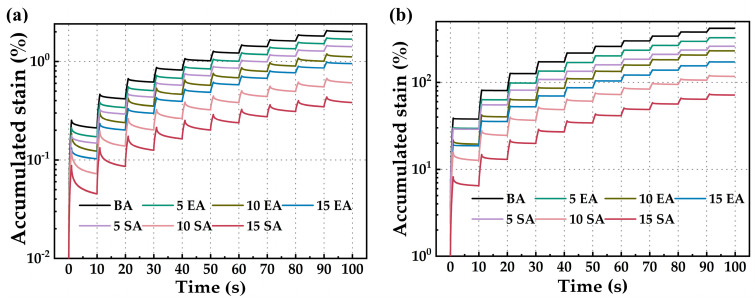
Creep under stress levels of 0.1 kPa (**a**) and 3.2 kPa (**b**) in the MSCR test of BA and GPMA.

Figure 11 displays the cumulative strain variation curves of various types of asphalt subjected to varying stress levels. It is evident that, irrespective of the stress level, the cumulative strain of asphalt gradually increases as the cyclic cumulative loading time increases. This indicates a gradual weakening of the resistance to permanent deformation of asphalt. Simultaneously, the progressive strain from high to low is as follows: BA > EUGMA > SEUGMA, suggesting that both SEUG and EUG can enhance the ability of BA to resist permanent deformation. Furthermore, the higher the contents of modifier used, the more pronounced the improvement effect. Due to the more extensive crosslinked network in the molecular structure of SEUG compared to EUG, SEUGMA has a higher proportion of elastic material. This leads to improved performance in recovering from high-temperature creep in SEUGMA.

To quantitatively assess the ability of GPMA to resist permanent deformation and its sensitivity to stress, the evaluation indexes *J*_nr_ and *R* were used. These indexes measure the permanent deformation resistance of asphalt under cyclic loading. A smaller *J*_nr_ and a larger *R* indicate a greater elastic restorative capacity of the asphalt [54]. The values of *J*_nr_ and *R* can be determined using Equations (9) and (10).

(9) $$J_{nr}=\frac{1}{10}\sum_{1}^{10}\frac{\varepsilon_{r}-\varepsilon_{0}}{\delta }$$

(10) $$R=\frac{1}{10}\sum_{1}^{10}\frac{\varepsilon_{p}-\varepsilon_{r}}{\varepsilon_{p}-\varepsilon_{0}}$$

where *ε*_0_ is the initial strain within per unit stress loading cycle; *ε_p_* is the peak strain within per unit stress loading cycle; *ε_r_* is the residual strain within per unit stress loading cycle; *δ* is the applied stress.

**Figure 12:**
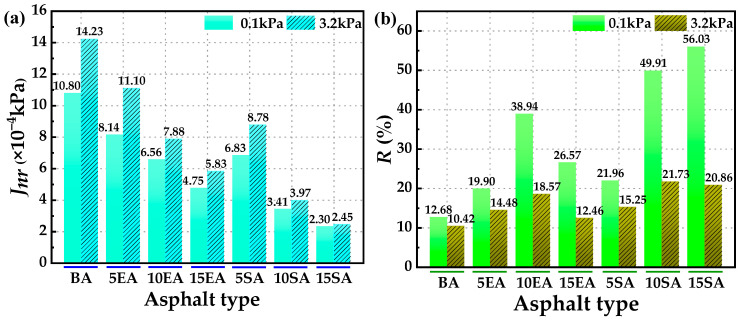
*J_nr_* and *R* of BA and GPMA: (**a**) *J_nr_* of BA and GPMA; (**b**) *R* of BA and GPMA.

Figure 12 displays the computed values of *J*_nr_ and *R*. Figure 12 clearly demonstrates that BA displays a higher level of unrecoverable creep flexibility and a lower average recovery rate when subjected to repeated loading. This indicates that BA has an inadequate elastic recovery capacity. The addition of GP leads to a gradual decrease in *J*_nr,0.1_ and *J*_nr,3.2_, while *R*_0.1_ and *R*_3.2_ show a gradual increase. This suggests that GP effectively enhances the resistance to permanent deformation and elastic recovery of the matrix asphalt. Furthermore, as the contents of GP increases, this enhancement becomes more evident. SEUGMA exhibits a lower *J*_nr_ and a higher *R* compared to EUGMA. This suggests that SEUGMA has higher resistance to permanent deformation and elastic recovery ability than EUGMA. The reason for this is the presence of a more intricate crosslinked network structure in SEUGMA.

**Figure 13:**
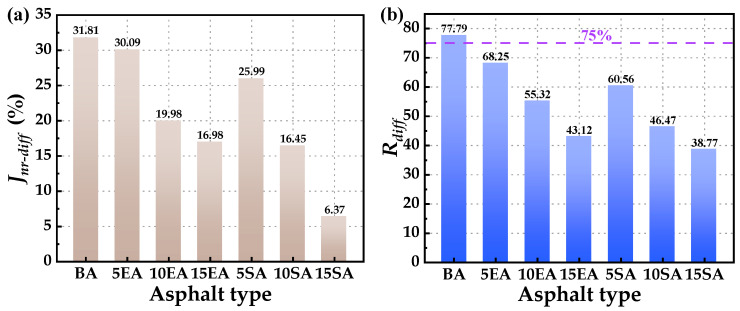
*J_nr-diff_* and *R_diff_* of BA and GPMA: (**a**) *J_nr-diff_* of BA and GPMA; (**b**) *R_diff_* of BA and GPMA.

To assess the stress sensitivity of asphalt, the two parameters of unrecoverable creep softness difference (*J_nr-diff_*) and creep recovery rate difference (*R_diff_*) were selected as the evaluation indexes. A lower *J_nr-diff_* and *R_diff_* indicate a lower level of stress sensitivity [55]. The calculations for *J_nr-diff_* and *R_diff_* can be derived using Equations (11) and (12). The settlement results for *J_nr-diff_* and *R_diff_* of various GPMA can be observed in Figure 13.

(11) $$J_{\mathrm{nr-diff}}=\frac{\left(J_{nr,3.2}-J_{\mathrm{nr}r,0.1}\right)}{J_{nr,0.1}}\times 100\%$$

(12) $$R_{diff}=\frac{\left(R_{0.1}-R_{3.2}\right)}{R_{0.1}}\times 100\%$$

Figure 13 clearly demonstrates that GPMA exhibits smaller *J_nr-diff_* and *R_diff_* compared to BA. Furthermore, the Rdiff value is below 75%, satisfying the criteria set by AASHTO MP 19-10 specification. Additionally, it is observed that as the GP contents increase, there is a corresponding decrease in *J_nr-diff_* and *R_diff_*. This suggests that GP has a beneficial impact on enhancing the stress sensitivity of asphalt. When comparing EUGMA and SEUGMA with the same content, it is observed that SEUGMA has smaller *J_nr-diff_* and *R_diff_*. This suggests that SEUGMA is more sensitive to stress and therefore performs better in terms of stress sensitivity. The presence of a more intricate crosslinked network structure in SEUG may explain this phenomenon. This structure acts as an elastic micro-skeleton, and the greater the amount of SEUG, the more pronounced its role as an elastic micro-skeleton becomes. Consequently, it enhances the ability of asphalt to resist high temperatures and frequent loading.

#### 4.2.3. Resistance to Cracking at Low Temperatures

**Figure 14:**
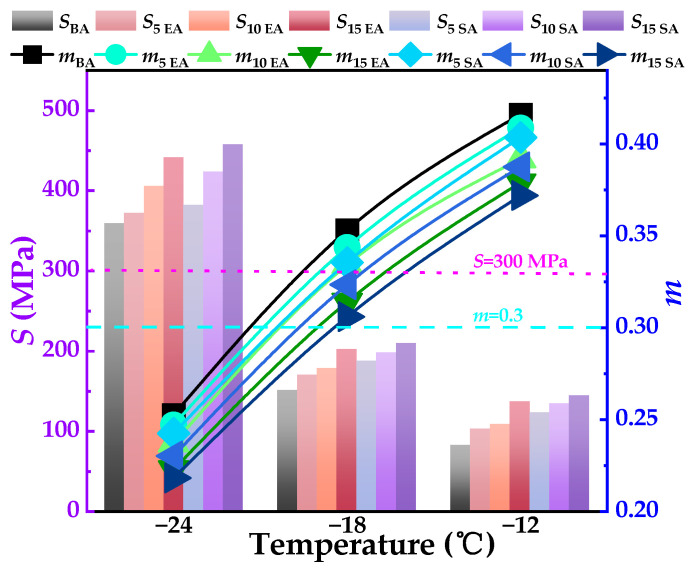
BBR test results of BA and GPMA.

This investigation aimed to assess the creep performance of asphalt in a low-temperature environment. The creep strength modulus (*S*) and creep rate (*m*) of asphalt were determined using the BBR test. The creep stiffness (*S*) indicates the capacity of asphalt to withstand loading, while the creep rate (*m*) represents the rate at which the asphalt’s strength changes. The low-temperature creep performance of asphalt improves as the *S* becomes smaller and the *m* becomes larger. Figure 14 displays the test results. Figure 14 illustrates that within the temperature range of −24 °C to −12 °C, the *S* of all asphalt samples increases as the temperature decreases, while the m decreases. These findings suggest that the ability of asphalt to resist cracking at low temperatures worsens as the temperature drops. The reason for this is that asphalt, being a material that is sensitive to temperature, can become brittle when exposed to low temperatures [56].

Under the same temperature conditions, the *S* are, in descending order, SEUGMA > EUGMA > BA. The m are, in descending order, BA > EUGMA > SEUGMA, indicating that both EUG and SEUG adversely affect the low-temperature cracking resistance of asphalt, and the larger the amount of the modifier doped, the more pronounced the adverse effect, and the adverse effect of SEUG is more serious than that of EUG on the low-temperature cracking resistance of asphalt. SEUG has a more serious adverse effect on the low-temperature cracking resistance of asphalt than EUG. This may be due to the fact that both EUG and SEUG absorb the light components of asphalt and undergo a swelling reaction, resulting in changes in the volume of asphalt, which in turn triggers the prolongation of the stress relaxation time of asphalt and reduces the low-temperature cracking resistance of asphalt. In addition, due to the poor plasticity of SEUG compared to EUG, this results in SEUGMA being less resistant to plastic deformation than EUGMA during low-temperature creep.

### 4.3. Modified Mechanism of GPMA

#### 4.3.1. FTIR Test

**Figure 15:**
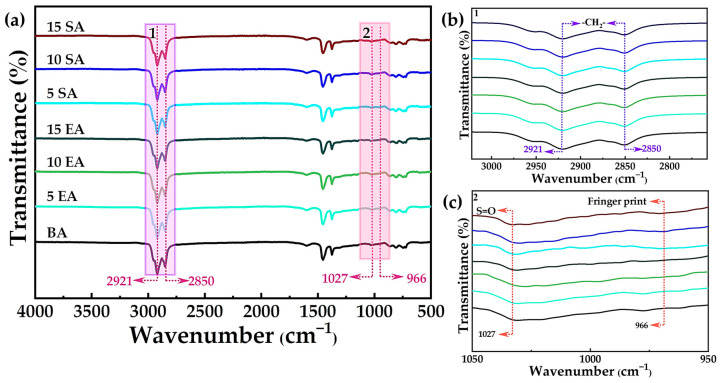
The infrared spectra of BA and GPMA: (**a**) infrared spectra in the range of 4000 cm^−1^ to 500 cm^−1^; (**b**) infrared spectra in the range of 3022 cm^−1^ to 2758 cm^−1^; (**c**) infrared spectra in the range of 1050 cm^−1^ to 950 cm^−1^.

To assess the impact of GP on the chemical composition and functional groups of BA, this investigation conducted a comprehensive analysis using infrared spectroscopic tests. The findings are presented in Figure 15. Figure 15 demonstrates that the infrared spectra of all asphalts were fundamentally alike, suggesting that the primary physical alterations took place during the preparation process for EUGMA and SEUGMA. The absorption peaks at 2921 cm^−1^ and 2850 cm^−1^ in BA are caused by the vibration of the -CH_2_ groups. These peaks are also the most prominent in the absorption spectrum of matrix bitumen. This indicates that EUG and SEUG absorb the saturated hydrocarbons in the bitumen through a solvation reaction, leading to the depletion of saturated hydrocarbons. The absorption peaks at 1027 cm^−1^ are caused by the vibration of the *S*=O group. The absorption peaks of EUGMA and BA almost coincide in the *S*=O absorption peak, but there is a significant difference between the absorption peaks of SEUGMA and BA. The intensity of the absorption peak deviation increases as the SEUGMA content increases. This suggests that SEUGMA increases the sulfur content of asphalt, possibly due to its susceptibility to desulfurization and degradation reactions under high-temperature and high-shear conditions. This phenomenon can be attributed to the susceptibility of SEUG to desulfurization degradation reaction under conditions of high temperature and high-speed shear. Consequently, the resulting *S* undergoes further reactions with O to form *S*=O groups. Furthermore, when compared to BA and EUGMA, SEUGMA exhibited a distinct absorption peak at 966 cm^−1^, suggesting the possibility of a chemical reaction taking place during the preparation of SEUGMA. This reaction likely resulted in the formation of a new functional group, which aligns with the observations made by Li et al. [21].

#### 4.3.2. FM Test

**Figure 16:**
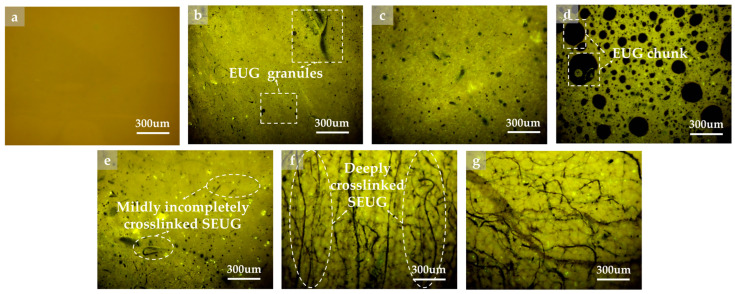
Different FM images of BA and GPMA: (**a**) FM image of BA; (**b**) FM image of 5wt% EUGMA; (**c**) FM image of 5wt% EUGMA; (**d**) FM image of 15wt% EUGMA; (**e**) FM image of 5wt% SEUGMA; (**f**) FM image of 10wt% SEUGMA; (**g**) FM image of 15wt% SEUGMA.

This investigation observed the behavior of BA and GPMA using FM, with a magnification of 300×. The findings are presented in Figure 16. Figure 16 demonstrates that the asphalt phase does not exhibit fluorescence under FM, but instead appears as a dark-yellow color. In the GPMA system, the asphalt phase functions as the continuous phase, while the GP phase acts as the dispersed phase. The GP is present in the form of black fluorescent dots or bands.

For 5wt% EUGMA, EUG is present as small and scattered black fluorescent dots. For 10wt% EUGMA, both the quantity and size of fluorescent dots exhibited an increase. For 15wt% EUGMA, the black fluorescent dots displayed noticeable aggregation. At this point, there is evidence that the distribution of EUG in asphalt deteriorates, potentially causing a decline in the storage stability of asphalt.

For 5wt% SEUGMA, black fluorescent dots and a small number of bands became visible. However, for 10wt% SEUG, the number of black fluorescent dots decreas, while the number of black bands gradually increased. The 15 wt% SEUGMA was observed to increase and form a more intricate crosslinked network structure. This could have a negative impact on the flexibility of the bitumen as a result of excessive reinforcement. The results indicate that asphalt with a 10wt% SEUGMA content exhibits the most significant swelling effect. Additionally, SEUG has the ability to optimize the reinforcing effect, enhance the resistance to elastic deformation of the asphalt, and prevent the formation of microcracks, thereby improving the durability of the asphalt.

#### 4.3.3. SEM Test

**Figure 17:**
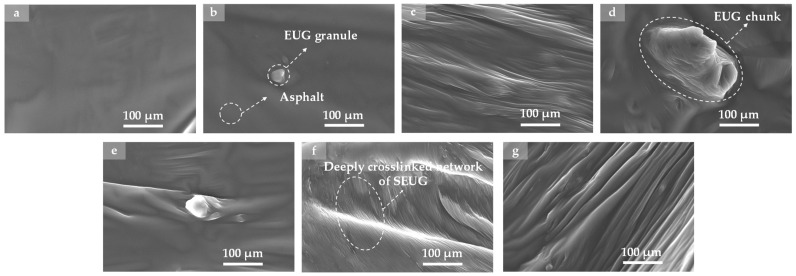
SEM images of BA and GPMA: (**a**) SEM image of BA; (**b**) SEM image of 5wt% EUGMA; (**c**) SEM image of 10wt% EUGMA; (**d**) SEM image of 15wt% EUGMA; (**e**) SEM image of 5wt% SEUGMA; (**f**) SEM image of 10wt% SEUGMA; (**g**) SEM image of 15wt% SEUGMA.

This investigation applied scanning electron microscopy (SEM) to observe the impact of GP on the micro-morphology of asphalt. The findings are presented in Figure 17. Figure 17 illustrates that the surface of BA is comparatively even, whereas GPMA exhibits greater microtexture and granular characteristics. The addition of GP alters the microscopic arrangement of asphalt and enhances the unevenness of its surface, potentially leading to beneficial enhancements in asphalt properties, including adhesion, resistance to cracking, and overall durability.

Figure 17b–d show that in EUGMA, EUG exists mainly in the form of particles, which are encapsulated by asphalt, exhibiting the characteristics of a non-homogeneous structure. The SEM images of 5wt% EUGMA show that the surface of the samples is relatively flat, with only a small amount of EUG particles dispersed in the asphalt. When the EUG content was increased to 10wt%, the EUG particles were more uniformly encapsulated by the asphalt, and the surface of 10wt% EUGMA appeared to be characterized by a homogeneous wrinkled structure. When the EUG content was increased to 15wt%, the EUG particles appeared to be significantly agglomerated, forming bumps and depressions of different sizes.

Figure 17e–g show that more folds and grooves as well as SEUG particles of different sizes appeared on the surface of SEUGMA compared to EUGMA, which exhibited obvious non-homogeneous structure characteristics. For 5wt% SEUGMA, a small number of folds and particles of different sizes appeared on the sample surface. For 10wt% SEUGMA, the sample surface exhibits smooth and uniform folds, and no obvious granular structure is seen. For 15wt% SEUGMA, the surface of the specimen showed a large number of irregularly shaped folds and granular structures. It can be seen that 10wt% SEUGMA has the best uniformity, which is due to the moderate amount of SEUG doping, in the preparation process, due to the rapid friction between the rotor and stator of the shear machine that generates a large amount of kinetic and thermal energy, so that part of the SEUG depolymerization occurs, and the coarser SEUG particles are ground into fine particles, which promotes the diffusion of the SEUG into the asphalt. SEUG is distributed in asphalt in the form of uniform bands, increasing the contact area between SEUG and asphalt, and the modification effect of SEUG is better transferred to asphalt, improving asphalt elasticity and crack resistance.

#### 4.3.4. AFM Test

**Figure 18:**
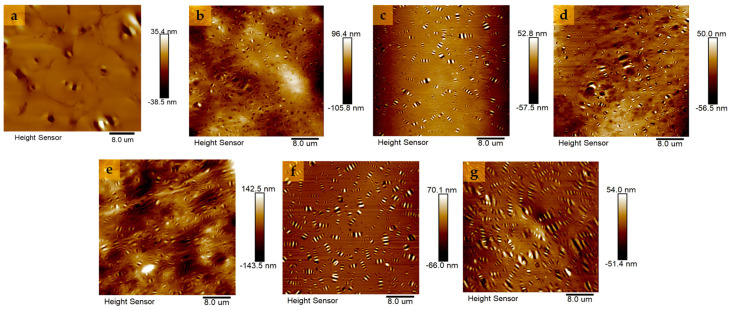
Two-dimensional AFM microscopic morphology of BA and GPMA: (**a**) AFM image of BA; (**b**) AFM image of 5wt% EUGMA; (**c**) AFM image of 10wt% EUGMA; (**d**) AFM image of 15wt% EUGMA; (**e**) AFM image of 5wt% SEUGMA; (**f**) AFM image of 10wt% SEUGMA; (**g**) AFM image of 15wt% SEUGMA.

In this research, the microstructures of different asphalt specimens were observed using the AFM technique, and the AFM 2D scanned images are shown in Figure 18. As can be seen in Figure 18, the AFM morphology of BA shows “honeycomb structures” of varying sizes, dispersed, and blurred morphology. The “bee-like structure” is considered to be a wax crystalline structure with an asphaltene core, with the white area representing hard asphaltene and the black area representing soft asphaltene [57]. There are more “bee-like structures” and clearer morphology in GPMA than in BA. Especially in SEUGMA, the number and area of “bee-like structures” are the largest, and even the phenomenon of superposition occurs. This indicates that the swelling of GP in asphalt led to a reorganization of BA components, an increase in heavy components, and a decrease in light components, which improved the high-temperature performance of asphalt and reduced the low-temperature performance of asphalt, and the 15wt% SEUG had the strongest swelling ability, which was consistent with the conclusion of the rheological test.

**Figure 19:**
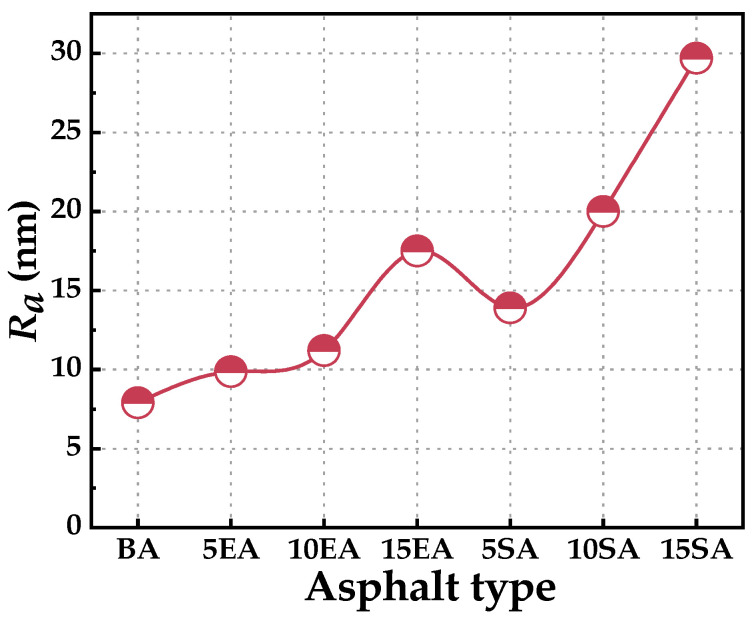
The roughness of BA and GPMA.

Figure 19 presents the variation pattern of root mean square roughness (*R_a_*) for different GPMA. It has been demonstrated that higher *R_a_* values in asphalt correspond to better adhesion [58]. The descending order of *R_a_* for three types of asphalt is as follows: SEUGMA > EUGMA > BA. This indicates that BA has the smoothest surface and the weakest adhesion, but the best resistance to deformation at low temperatures. SEUGMA has the roughest surface, the strongest adhesion, and the strongest stability at high temperatures, but the poorest resistance to deformation at low temperatures. This aligns with the conclusion drawn from the macroscopic high-temperature rheological performance test.

## 5. Conclusions

This investigation comprehensively assessed the influence of GP as a modifier on asphalt properties and elucidated the mechanism of GP modifies asphalt. The following conclusions were drawn:

(1) The MD results indicate that the compatibility between EUG and asphalt is superior, and the compatibility with BA achieves its optimum at a temperature of 165 °C. EUG forms a micellar system with asphaltenes and the saturates of asphaltene by polar interactions. SEUG is not compatible with asphalt; however, the compatibility between SEUG and asphalt is optimal at a temperature of 180 °C. SEUG forms π-π conjugation interactions with naphthene aromatics and polar aromatics present in asphaltene. The primary source of interaction energy between GP and asphalt molecules is predominantly derived from van der Waals forces.
(2) The optimal preparation process of GPMA was discussed by using the gray correlation theory, and the results show that the optimal preparation process parameters of EUGMA were T_1_ = 145 °C, T_2_ = 165 °C; S_1_ =3000 r/min, S_2_ = 5000 r/min; and t_1_ = 60 min, t_2_ = 60 min. The optimal preparation process parameters of SEUGMA were T_1_ = 155 °C, T_2_ = 180 °C; S_1_ = 4000 r/min, S_2_ = 6000 r/min; and t_1_ = 90 min, t_2_ = 90 min. Since the T_2_ of EUGMA and SEUGMA are consistent with the results of MD simulation, the validity of MD simulation is proved.
(3) The macro rheological test findings indicate that both EUG and SEUG have a considerable positive impact on the high-temperature stability and deformation resistance of asphalt. However, they have a negative effect on the low-temperature performance of asphalt. The 15wt% SEUGMA has superior high-temperature stability but poor resistance to low-temperature cracking. By conducting a microscopic performance test, it was determined that the preparation method of EUGMA involved physical blending, while the preparation method of SEUGMA primarily involved physical blending with a minor component of chemical mixing.

In summary, GP as a new asphalt modifier shows advantages in enhancing the high-temperature performance of asphalt. Future research will focus on improving the insufficient low-temperature performance of GPMA and exploring multiscale composite modification strategies to achieve comprehensive optimization across different temperature intervals.

## Figures and Tables

**Table 1:** Simulated versus measured results of thermodynamic parameters.

Molecular Models	Criteria	MD Results	EL	Errors (%)
EUG	*ρ* (g·cm^−3^)	0.882	0.91~0.98 [38]	3.3
	*δ*_Hansen_ (g·cm^−3^)^1/2^	16.53	16.2~17.0 [23,39]	2~2.8
SEUG	*ρ* (g·cm^−3^)	0.984	1.009	2.5
	*δ*_Hansen_ (g·cm^−3^)^1/2^	11.041	-	-
BA	*ρ* (g·cm^−3^)	0.998	0.968~1.034 [40,41]	4.1~4.9
	*δ*_Hansen_ (g·cm^−3^)^1/2^	17.802	13.30~22.50 [42]	1.0
5wt% EUGMA	*ρ* (g·cm^−3^)	1.004	1.037	3.2
10wt% EUGMA	*ρ* (g·cm^−3^)	1.006	1.042	3.5
15wt% EUGMA	*ρ* (g·cm^−3^)	1.007	1.049	4.0
5wt% SEUGMA	*ρ* (g·cm^−3^)	1.024	1.057	3.1
10wt% SEUGMA	*ρ* (g·cm^−3^)	1.028	1.062	3.2
15wt% SEUGMA	*ρ* (g·cm^−3^)	1.032	1.067	3.3

Note: EL represents experiment or the literature results.

**Table 2:** Technical specifications of base asphalt.

Property		Result	Specification Limits	Testing Method [43]
Penetration (25 °C, 0.1 mm)		90.5	80~100	T0604—2011
Softening point (R&B, °C)		47.7	≥40	T0606—2011
Ductility (5 °C, cm)		9.5	-	T0605—2011
RTFOT Residuum	Mass loss rate (%)	0.05	≤±0.8	T0610—2011
	Penetration ratio (25 °C, %)	61.1	≥57	T0610—2011
	Ductility (5 °C, cm)	8.2	≥8	T0610—2011

**Table 3:** The orthogonal experimental design of GPMA.

Asphalt Type	Sample Number	Shear Temperature (°C)	Shear Rate (r/min)	Shear Time (min)
EUGMA	EA1	*T*_1_: 130; *T*_2_:150	*S*_1_: 3000; *S*_2_: 3000	*t*_1_: 40; *t*_2_: 40
	EA2	*T*_1_: 130; *T*_2_:150	*S*_1_: 3000; *S*_2_: 5000	*t*_1_: 40; *t*_2_: 60
	EA3	*T*_1_: 130; *T*_2_:150	*S*_1_: 5000; *S*_2_: 5000	*t*_1_: 60; *t*_2_: 60
	EA4	*T*_1_: 145; *T*_2_:165	*S*_1_: 3000; *S*_2_: 3000	*t*_1_: 40; *t*_2_: 60
	EA5	*T*_1_: 145; *T*_2_:165	*S*_1_: 3000; *S*_2_: 5000	*t*_1_: 60; *t*_2_: 60
	EA6	*T*_1_: 145; *T*_2_:165	*S*_1_: 5000; *S*_2_: 5000	*t*_1_: 40; *t*_2_: 40
	EA7	*T*_1_: 160; *T*_2_:180	*S*_1_: 3000; *S*_2_: 3000	*t*_1_: 60; *t*_2_: 60
	EA8	*T*_1_: 160; *T*_2_:180	*S*_1_: 3000; *S*_2_: 5000	*t*_1_: 40; *t*_2_: 40
	EA9	*T*_1_: 160; *T*_2_:180	*S*_1_: 5000; *S*_2_: 5000	*t*_1_: 40; *t*_2_: 60
SEUGMA	SA1	*T*_1_: 140; *T*_2_:165	*S*_1_: 4000; *S*_2_: 4000	*t*_1_: 60; *t*_2_: 90
	SA2	*T*_1_: 140; *T*_2_:165	*S*_1_: 4000; *S*_2_: 6000	*t*_1_: 90; *t*_2_: 60
	SA3	*T*_1_: 140; *T*_2_:165	*S*_1_: 6000; *S*_2_: 6000	*t*_1_: 90; *t*_2_: 90
	SA4	*T*_1_: 155; *T*_2_:180	*S*_1_: 4000; *S*_2_: 4000	*t*_1_: 90; *t*_2_: 60
	SA5	*T*_1_: 155; *T*_2_:180	*S*_1_: 4000; *S*_2_: 6000	*t*_1_: 90; *t*_2_: 90
	SA6	*T*_1_: 155; *T*_2_:180	*S*_1_: 6000; *S*_2_: 6000	*t*_1_: 60; *t*_2_: 90
	SA7	*T*_1_: 170; *T*_2_:195	*S*_1_: 4000; *S*_2_: 4000	*t*_1_: 90; *t*_2_: 90
	SA8	*T*_1_: 170; *T*_2_:195	*S*_1_: 4000; *S*_2_: 6000	*t*_1_: 60; *t*_2_: 90
	SA9	*T*_1_: 170; *T*_2_:195	*S*_1_: 6000; *S*_2_: 6000	*t*_1_: 90; *t*_2_: 60

**Table 4:** Calculation results of correlation coefficients and degrees of correlation for various indicators.

Sample Number	$\xi_{i}(k)$				$\gamma_{i}(k)$
	Penetration (0.1 mm)	Softening Point (°C)	Ductility (cm)	High-Temperature Grading Temperature (°C)	
EA 1	0.356	0.374	0.333	0.580	0.411
EA 2	0.376	0.385	0.368	0.621	0.438
EA 3	0.451	0.415	0.402	0.724	0.498
EA 4	0.600	0.498	0.471	0.897	0.616
EA 5	1.000	0.636	1.000	1.000	0.909
EA 6	0.705	0.686	0.580	0.963	0.734
EA 7	0.550	0.695	0.447	0.929	0.655
EA 8	0.488	0.868	0.443	0.840	0.660
EA 9	0.467	1.000	0.412	0.868	0.687
SA 1	0.544	0.333	0.462	0.570	0.477
SA 2	0.570	0.360	0.544	0.605	0.520
SA 3	0.605	0.392	0.605	0.620	0.556
SA 4	0.721	0.521	0.662	0.891	0.699
SA 5	0.803	0.570	0.778	1.000	0.788
SA 6	1.000	0.695	1.000	0.961	0.914
SA 7	0.731	0.721	0.790	0.690	0.733
SA 8	0.645	0.891	0.721	0.662	0.730
SA 9	0.538	1.000	0.645	0.645	0.707

## Data Availability

The original contributions presented in the study are included in the article, further inquiries can be directed to the corresponding authors.

## Abbreviations

gutta-percha, GP; gutta-percha-modified asphalt, GPMA; eucommia ulmoides gum, EUG; sulfur-vulcanized eucommia ulmoides gum, SEUG; EUG-modified asphalt, EUGMA; sulfur-vulcanized eucommia ulmoides gum-modified asphalt, SEUGMA; styrene–butadiene–styrene block copolymer, SBS; base asphalt, BA; repeating unit technique, RUM; canonical ensemble, NVT; constant pressure and temperature, NPT; 5wt% (10wt% and 15wt%) EUGMA and 5 (10 and 15) EA; 5wt% (10wt% and 15wt%) SEUGMA and 5 (10 and 15) SA; represents experiment or results from the literature, EL; dynamic shear rheology, DSR; multiple stress creep recovery, MSCR; low-temperature bending beam rheology, BBR; Fourier transform infrared spectroscopy, FTIR; fluorescence microscopy, FM; scanning electron microscope, SEM; atomic force microscopy, AFM; asphaltenes, AS; polar aromatics, RE; naphthene aromatics, AC; saturates, SA; cohesive energy density, CED.
